# Notes and News

**Published:** 1900-12-08

**Authors:** 


### NOTES AND NEWS.

The Queen has granted permission to Sir William MacCormac to wear the Cross of Commander of the Legion of Honour of France awarded to him for services to the wounded in the Franco-Prussian War, and to the International Medical Congress held in Paris this year.

The irony of fate has willed it that the British Medical Association, which a few years ago fought fiercely against the admission of ladies to its membership, is to open the new century by holding its annual meeting in a ladies' college—Cheltenham to wit.

The City of London Hospital for Diseases of the Chest, Victoria Park, is appealing for a new organ in the chapel. The present one has been in use for forty years. The amount now required is only £20, the rest of the total sum necessary, namely £250, having been secured.

A Marine Ambulance Corps, composed from amongst those employed at the docks, has been established at Bristol. All members must hold St. John's Ambulance First Aid Certificates, and hold themselves ready to pass an examination once a year. This is the first marine ambulance in the kingdom: it has a most useful work before it, and has been instituted and organised on excellent lines.

The British Congress on Tuberculosis in London will be opened by the Prince of Wales on Monday, July 22, 1901, and will be continued on the following days of that week. The Congress will have four sections:—Pathology, Medicine, State Medicine, and Veterinary Medicine, which will meet on the mornings of Tuesday, Wednesday, Thursday and Friday, and Saturday will be devoted to excursions. Further particulars can be obtained from the Honorary Secretary General, 20 Hanover Square. The subscription for members of the Congress will be £1, which will include a copy of the "Transactions." A fund is being formed to defray the expenses connected with the Congress, which must be considerable. It already amounts to £800, but at least five times this amount will be required.

Although the formal opening ceremony of the Jubilee wing at the Nottingham General Hospital is postponed for the present, the wards are to come into use at once.

As the Belfast Board of Guardians have decided to erect a new nurses' home at their infirmary, the present building used for the purpose is to be converted into a children's hospital.

Mrs. Medley, of Winsford Tower, Beaworthy, has erected a cottage hospital for the use of the poor of the district. The hospital overlooks Dartmoor, and is situated close to Halwill Junction Station. It contains four wards for two beds in each, a surgery, consulting room, operating room, and the usual offices. The whole equipment is up to date, and in fact rather in advance of that provided in most cottage hospitals. The total cost is estimated at about £16,000.

Sir Christopher Nixon, in his address on the Relation of Veterinary Science to Human Medicine before the Irish Royal Academy of Medicine, laid stress upon the need of a Jenner Institute in Ireland. He pointed out that both preventative and curative medicine must look towards bacteriology as the direction in which to seek for progress. He deplored the apathy of the State towards scientific progress, and held it in some measure responsible for the backward position of Great Britain compared with some foreign countries as regards physiology, pathology, and chemistry.

The Sanitary Institute passed resolutions at their Conference on the Housing of the Working Classes which declared that the Conference was of opinion that the present method of dealing with insanitary areas is extravagant and unsatisfactory, and it urged Parliament to provide some less expensive and more expeditious mode of procedure. Further, that in the opinion of the Conference the Local Government Board should at once extend the period of repayment actually granted to local authorities for building loans under Part 3 of the Housing of the Working Classes Act of 1890, to the full statutory period of sixty years, and that they should relax and vary the restrictions on building cottages, especially in the direction of enabling more rooms to be provided at a less cost per room where this can be done without prejudice to considerations of sanitation and safety. It also was decided that Parliament should be asked to extend the period for the repayment of housing loans to 100 years in the case of land, and facilitate the advance of money by the Public Works Loan Commissioners at the market rate of interest for the purpose of housing the working classes. The resolutions were sent to the members of Her Majesty's Government.

The interim report issued by Sir Landon Brunton, Dr. Stevenson, Mr. Gordon Salmon, Dr. Luff, and Dr. Buckley, the experts appointed by the Manchester Brewers' Association to inquire into the alleged presence of arsenic in beer, fully bears out what we said on the subject last week. They trace the mischief to certain materials supplied by a certain firm, and they recommend that all beer in the brewing of which this substance has been used should be recalled, and if found contaminated should be at once destroyed, and that no beer should be sent out until it has been tested and shown to be free from arsenic.

